# Identification of an adipose tissue-resident pro-preadipocyte population

**DOI:** 10.1016/j.celrep.2023.112440

**Published:** 2023-04-27

**Authors:** Min Chen, Soochi Kim, Liang Li, Sourav Chattopadhyay, Thomas A. Rando, Brian J. Feldman

**Affiliations:** 1 Department of Pediatrics, University of California, San Francisco School of Medicine, San Francisco, CA 94158, USA; 2 Department of Neurology and Neurological Sciences, Stanford University School of Medicine, Stanford, CA 94305, USA; 3 Broad Stem Cell Research Center, University of California Los Angeles, Los Angeles, CA 90095, USA; 4 Nutrition and Obesity Research Center, University of California, San Francisco, San Francisco, CA 94158, USA; 5 Lead contact

## Abstract

Elucidating the transitional stages that define the pathway stem cells progress through during differentiation advances our understanding of biology and fosters the identification of therapeutic opportunities. However, distinguishing progenitor cells from other cell types and placing them in an epistatic pathway is challenging. This is exemplified in the adipocyte lineage, where the stromal vascular fraction (SVF) from adipose tissue is enriched for progenitor cells but also contains heterogeneous populations of cells. Single-cell RNA sequencing (scRNA-seq) has begun to facilitate the deconvolution of cell types in the SVF, and a hierarchical structure is emerging. Here, we use scRNA-seq to discover a population of CD31^−^ CD45^−^ cells in the SVF that are distinguished by a specific expression profile. Further, we place this population on an epistatic pathway upstream of the previously defined preadipocyte population. Finally, we discover functional properties of this population with broad implications, including revealing physiological mechanisms that regulate adipogenesis.

## In brief

Chen et al. identify a distinct population of adipose progenitor cells, which is upstream in an epistatic pathway with preadipocytes, in white adipose tissue. Using both primary mouse and human adipose cells, they reveal that *Noct* is a defining marker with functional effects on these cells, which they term pro-preadipocytes.

## Graphical Abstract

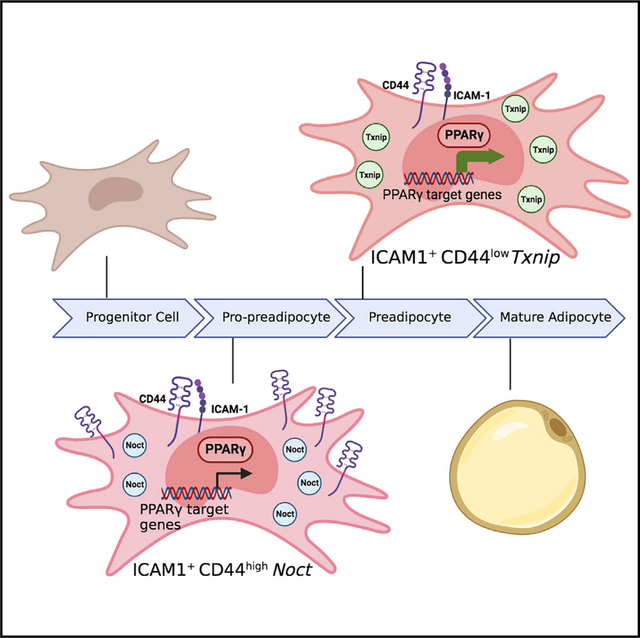

## INTRODUCTION

Mature adipocytes have diverse and critical roles in systemic physiology and are implicated in the pathogenesis of diseases, including obesity and diabetes.^1,2^ Furthermore, regulation of the formation of new adipocytes (adipogenesis) is responsive to systemic signals, including changes in diet and hormone levels, and dysfunctional adipogenesis leads to disease.^3^ In addition to medical and physiological implications, adipogenesis also provides a relevant system to elucidate basic mechanisms of stem cell biology.^4^ Importantly, a significant amount of adipogenesis occurs postnatally, facilitating observation and study of the process at the cellular and molecular levels in primary cells.^5–7^ In addition, adipocyte precursor cells reside in the adipose tissue depots themselves, making isolation and investigation of primary adipocyte precursor cells feasible.^8,9^

Adipogenesis can be induced in tissue culture in either primary precursor cells or surrogate model cell lines with differentiation potential.^10^ The ease with which induction of adipogenesis can be achieved in tissue culture has led to significant advances in our understanding of the molecular cascade that directs this process.^11^ However, it could be argued that the simplicity with which surrogate cell lines can be induced to undergo adipogenesis has delayed advancing our understanding of bona fide adipocyte progenitor cell biology. Historically, attempts at progress in this area have focused on using primary stromal vascular fractions (SVFs) isolated from adipose depots, which are enriched in progenitor cells that can be induced to undergo adipogenesis. However, the SVF is heterogeneous and likely contains multiple distinct types of adipocyte progenitor populations.^12^ Studies using defined surface markers have refined our definitions of more homogeneous subpopulations of cells isolated from the SVF that meet stricter definitions of progenitor cells, notably advancing the field.^13^ More recently, the application of next-generation single-cell RNA sequencing (scRNA-seq) has revealed important details and a hierarchy of different progenitor populations that exist within the SVF.^14–16^

Here, we report the results of scRNA-seq of CD31^−^ CD45^−^ cells isolated from SVF. Using stringent bioinformatical gates, we elucidate subpopulations within the SVF that had not been previously distinguished. Further, we reveal that an ICAM1^+^ CD44^high^ population of cells precedes an ICAM^+^ CD44^low^ population during adipogenesis. We found that this ICAM1^+^ CD44^high^ population has low PPARγ signaling but is composed of cells in a necessary transitional state in the adipogenesis process. We introduce the term “pro-preadipocyte” to describe this population to recognize that our findings support that this population is required and precedes the preadipocyte population in an epistatic adipogenesis pathway.

## RESULTS

### Identification of a distinct subpopulation of adipocyte progenitor cells

**Figure 1.**
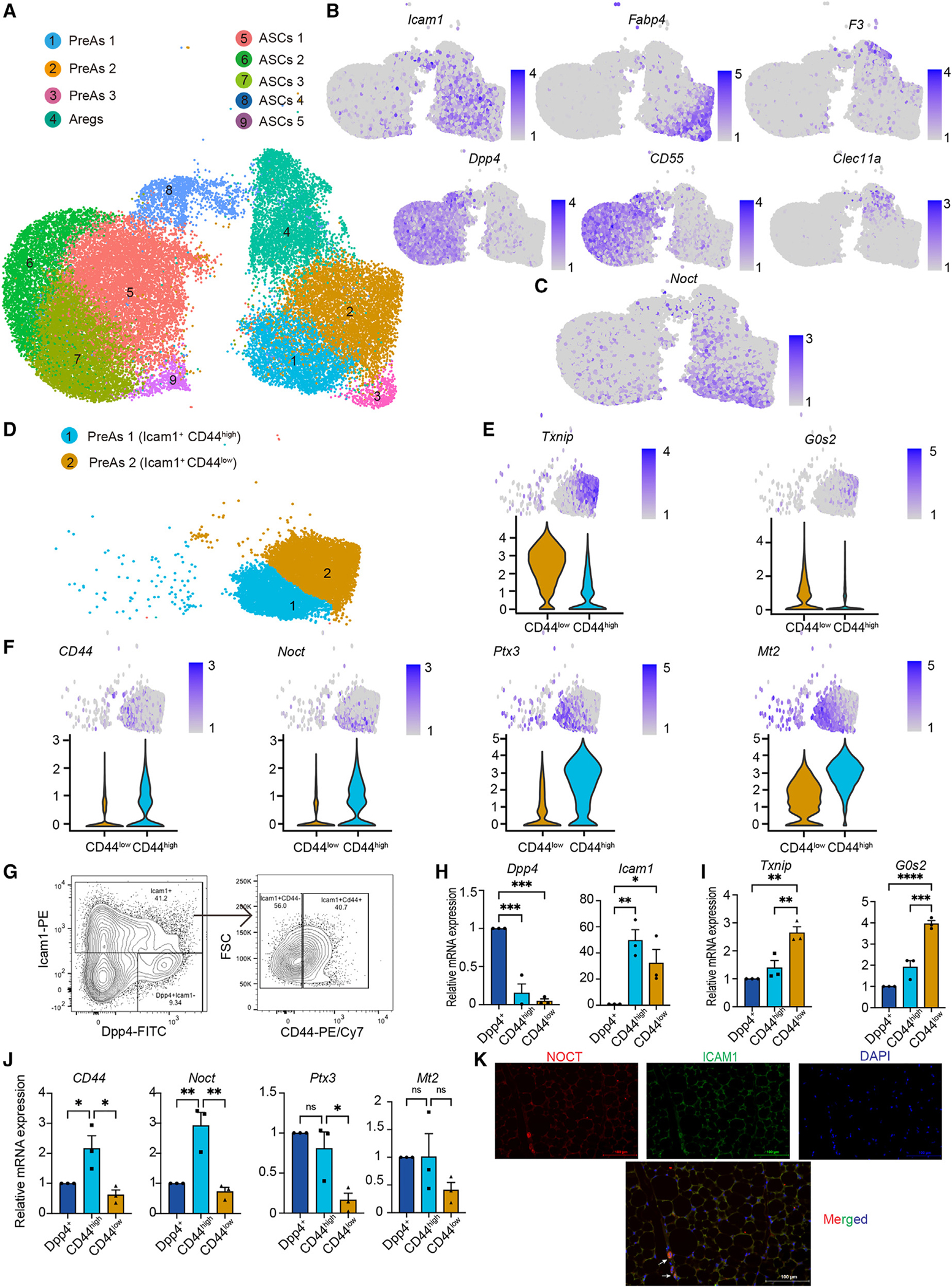
Identification of novel subpopulations within stromal vascular cells from subcutaneous white adipose depots (A) Uniform manifold approximation and projection (UMAP) of CD45^−^ CD31^−^ cells isolated from SVF reveals 9 distinct cell clusters. (B) Individual gene UMAP showing expression and distributions of known markers within this dataset. (C) Individual gene UMAP showing expression and distributions of *Noct* within this dataset. (D) UMAP map of CD44 and ICAM1 expression revealing distinct clusters. (E) UMAP feature and violin plots revealing additional markers expressed in ICAM1^+^ CD44^low^ cluster. (F) UMAP feature and violin plots revealing additional markers expressed in ICAM1^+^ CD44^high^ cluster. (G) Representative FACS plots showing gating for prospective separation and isolation of DPP4, ICAM1^+^ CD44^high^, and ICAM1^+^ CD44^low^ populations. Gates were set using one minus control methods (see Figure S1D). (H–J) qRT-PCR results quantifying the expression levels of marker genes in distinct populations prospectively isolated by FACS. qPCR cycle threshold (CT) values for each reaction were normalized against the expression of housekeeping gene Tbp in the same sample. Normalized results are displayed relative to the Dpp^+^ population. Significance was determined by ANOVA with a post hoc Dunnett’s correction. (K) Images from immunohistochemistry sections single channel (top) and merged (bottom) labeling identifies ICAM1^+^ NOCT cells (arrows) in subcutaneous inguinal white adipose tissue (iWAT). n = 3 mice were used for scRNA-seq and analysis, n = 4–5 mice were used for FACS and qRT-PCR experiments. Error bars represent ± SDs, *p < 0.05, **p < 0.01, ***p < 0.001, ****p < 0.0001.

Continued improvements in scRNA-seq technology enable more detailed insights into the adipocyte progenitor populations.^17^ To take advantage of these advances, we performed scRNA-seq of CD31^−^ CD45^−^ cells within the SVF from subcutaneous inguinal adipose depots (Figures S1A and S1B). When we applied similar bioinformatical analysis parameters to these data as others used,^14^ we verified our ability to detect previously described subpopulations of cells, including Dpp4^+^ adipocyte stem cells (ASCs), adipocyte regulatory cells (Aregs), and Icam1^+^ preadipocytes (PreAs) (Figures 1A and 1B). These results were reproducible across 3 biological replicates, validating our scRNA-seq datasets (Figure S1C). Of note, by increasing the resolution parameter in the analysis of our sequencing runs, we were intrigued that our studies also elucidated previously undescribed populations that separate the Icam1^+^ PreA subcluster into two distinct populations. Specifically, this analysis revealed that the previously described Icam1^+^ cluster^14^ segregates into two populations with distinct expression profiles, with the expression levels CD44, Nocturnin (Noct), thioredoxin-interacting protein (Txnip), G0/G1 switch 2 (G0s2), pentraxin 3 (Ptx3), and metallothionein 2 (Mt2) appearing as the most robust markers of these distinct subpopulations (Figures 1C–1F).

To prospectively test for the presence of these novel populations, we performed fluorescence-activated cell sorting (FACS) and isolated ICAM1^+^ CD44^high^ and ICAM1^+^ CD44^low^ cells and, for comparison, ICAM1^−^ DPP4^+^ cells from the CD31^−^ CD45^−^ population (Figures 1G, 1H, and S1D). We then used quantitative RT-PCR (qRT-PCR) to measure the expression levels of the putative subpopulation markers identified in these scRNA-seq analyses. These studies confirmed, in prospectively isolated cells, the presence of a population of cells that highly express *CD44, Noct, Ptx3*, and *Mt2* and that can be distinctly defined by expression levels from a population of cells that express low levels of those same markers and reciprocally selectively express high levels of the markers Txnip and G0s2 (Figures 1I and 1J).

We performed immunohistochemistry to attempt to locate these cells within the intact adipose depot. As anticipated, we found that the cells are a minority population within the fat pad. However, we were able to detect them often appearing in small clusters of 2–5 cells (Figure 1K).

**Figure 2.**
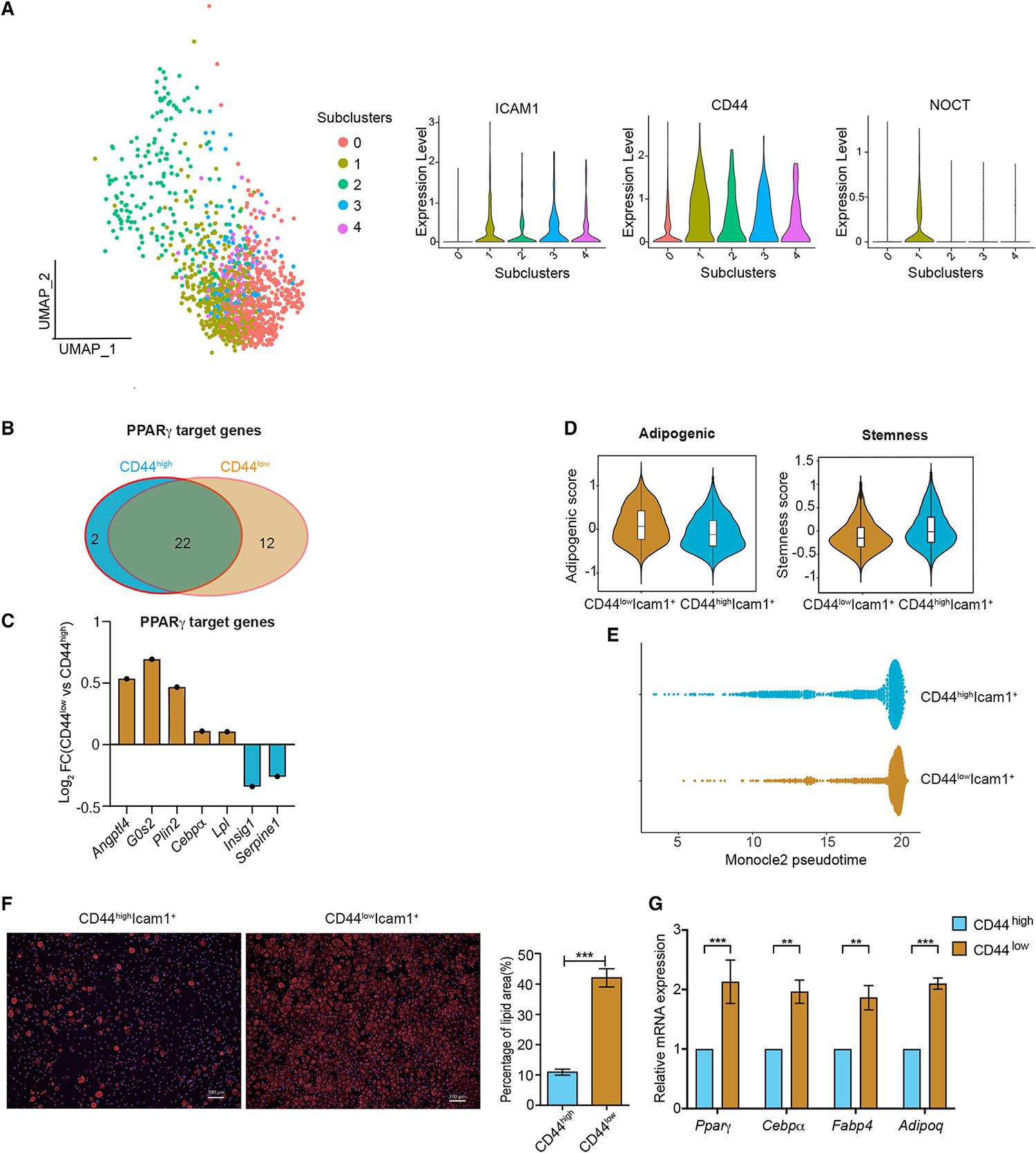
ICAM1^+^ CD44^high^ cells are pro-preadipocytes (A) (Left) UMAP of mesenchymal stem cells from *Tabula sapiens* fat tissue, with 5 distinct subclusters. (Right) Violin plots identifying a ICAM1^+^ CD44^high^ with NOCT expression population (subcluster 1). (B and C) ICAM1^+^ CD44^high^ population expresses fewer PPARγ target genes (B) and PPARγ target genes are expressed at lower levels (C). (D) The ICAM1^+^ CD44^high^ population has a lower adipogenic and higher stemness score than the ICAM1^+^ CD44^low^ population. (E) Pseudotime using Monocle 2 reveals a proximal relationship between ICAM1^+^ CD44^high^ and ICAM1^+^ CD44^low^ populations, with ICAM1^+^ CD44^high^ being earlier. (F) Detection of neutral lipids with BODIPY, marking differentiated adipocytes and quantification (performed on 4 or more images for each condition). Significance was determined by Student’s t test. (G) qRT-PCR quantifying the expression of markers of adipogenesis on cell postexposure to a minimal cocktail reveals that ICAM1^+^ CD44^low^ cells are more readily induced to differentiate than ICAM1^+^ CD44^high^ cells. Significance was determined by ANOVA with a post hoc Šidák correction. n = 4–5 mice were used in each independent FACS and qRT-PCR experiment. Each independent experiment was repeated at least 3 times. Error bars represent ± SDs, **p < 0.01, ***p < 0.001.

Next, we re-analyzed published publicly available scRNA-seq datasets to test if we could detect analogous populations despite experimental differences, including sequencing depth. Using the dataset published by Emont et al.,^18^ Icam1^+^ cells were subsetted and re-clustered with higher resolution, resulting in 6 distinct subclusters (Figure S2), including one subcluster (subcluster 5) that was Icam1^+^ CD44^high^ Noct expressing (Figure S2), analogous to the population we identified in our scRNA-seq experiments. Finally, we probed the published mesenchymal stem cell datasets from the single-cell transcriptomic atlas of humans.^19^ These data were subsetted and re-clustered at higher resolution. This data analysis resulted 5 distinct subclusters (Figure 2A). Of those, subcluster 1 is a distinct population of *ICAM1*^+^ *CD44*^high^ and *NOCT*-expressing cells (Figure 2A), suggesting that this distinct population is conserved in humans.

### ICAM1^+^ CD44^high^ *Noct*-expressing cells in the SVF are pro-PreAs

Next, we further compared the ICAM1^+^ CD44^high^ and ICAM1^+^ CD44^low^ populations bioinformatically. Intriguingly, while the levels of Pparγ expression itself are similar across the two populations, we discovered that PPAR signaling, as defined by modulation of downstream target genes, was significantly enriched in the ICAM1^+^ CD44^low^ *Txnip*-expressing population (Figures S3A, 2B, and 2C). This is notable because of the well-established role of PPAR signaling in adipogenesis, with increased signaling associated with and promoting differentiation.^4^ Therefore, this finding prompted us to hypothesize that the ICAM1^+^ CD44^low^ *Noct*-expressing population is both distinct and further advanced in the differentiation process than the ICAM1^+^ CD44^high^ population. To test this possibility, we calculated and compared the stemness and adipogenic scores^15,20^ of the populations. These studies revealed that the ICAM1^+^ CD44^high^ *Noct*-expressing population had a higher stemness score and a lower adipogenic score than the ICAM1^+^ CD44^low^ *Txnip*-expressing population (Figure 2D). We next used Monocle2^21^ to perform pseudotime trajectory analysis^21–23^ and test for temporal differences in the adipocyte lineage progression. The results of this analysis revealed that these populations are adjacent to each other in the adipocyte lineage progression, with the ICAM1^+^ CD44^high^ *Noct*-expressing population immediately preceding the ICAM1^+^ CD44^low^ *Txnip*-expressing population (Figure 2E).

Next, we empirically tested the functional implications of these distinctions. We hypothesized that cells further advanced in the adipocyte lineage progression would be more readily provoked to complete the progression through differentiation into mature adipocytes. Indeed, our studies demonstrated that a minimal differentiation cocktail that results in low level of adipogenesis in ICAM1^+^ CD44^high^ cells^14^ stimulated substantial levels of adipogenesis of ICAM1^+^ CD44^low^ cells, as assessed by both detection and quantification of the development of intracellular lipid droplets, a marker of mature adipocytes (Figure 2F) and qRT-PCR quantifying the expression levels of molecular markers of adipogenesis (Figure 2G).

**Figure 3.**
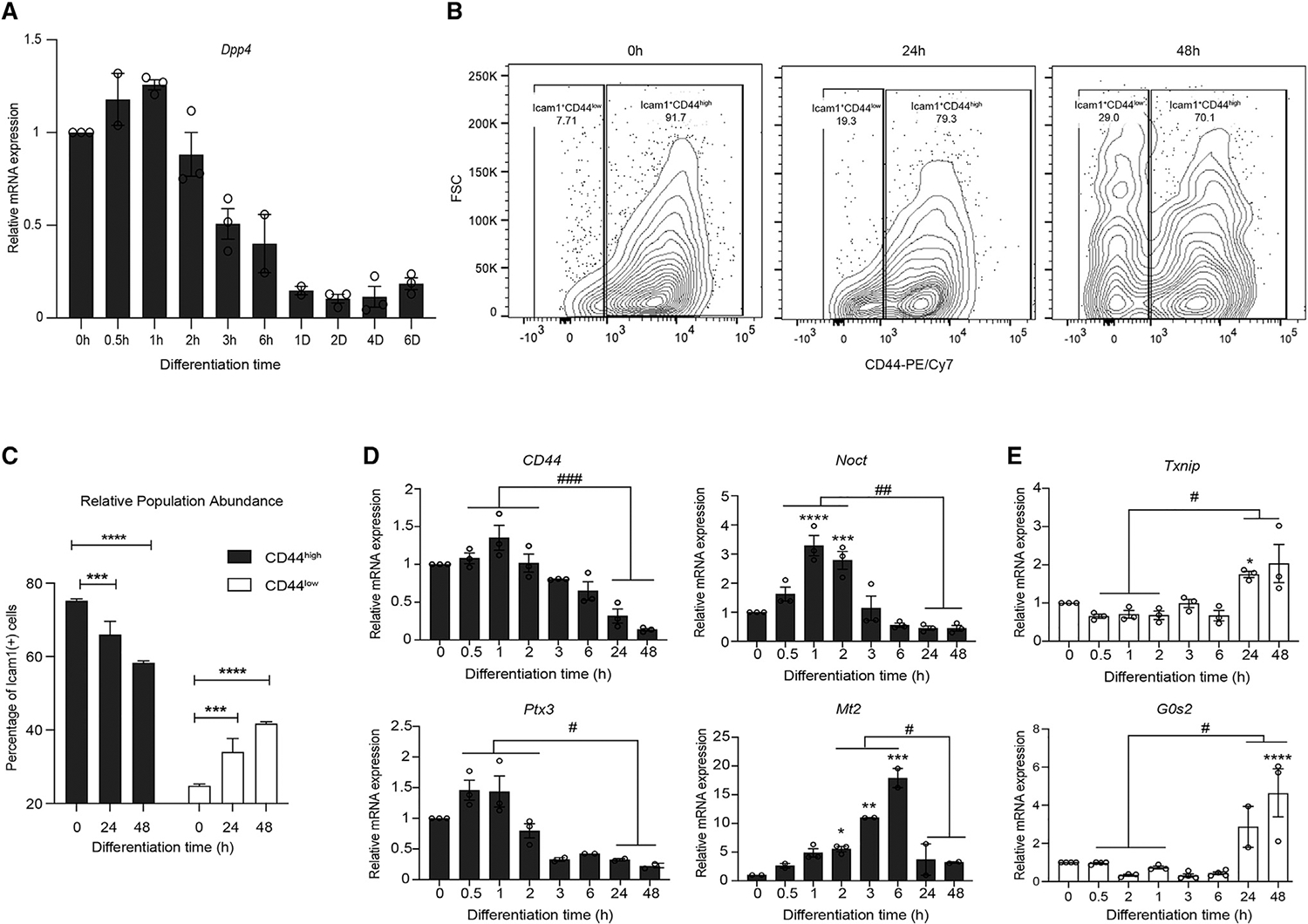
Induction of adipogenesis stimulates the transition of pro-preadipocytes into the ICAM1^+^ CD44^low^ population (A) qRT-PCR quantifying *Dpp4* expression levels postinduction of adipogenesis. (B) Representative flow cytometry plots monitoring the emergence of an ICAM1^+^ CD44^low^ population postinduction of adipogenesis. (C) Quantification of the transition from an abundant ICAM1^+^ CD44^high^ population to the emergence of the ICAM1^+^ CD44^low^ population. Significance was determined by ANOVA with a post hoc Šidák correction. (D) qRT-PCR quantifying expression markers of the ICAM1^+^ CD44^high^ population postinduction of adipogenesis. Significance was determined by ANOVA with a post hoc Dunnett’s correction. (E) qRT-PCR quantifying expression markers of the ICAM1^+^ CD44^low^ population postinduction of adipogenesis. Significance was determined by ANOVA with a post hoc Dunnett’s correction. n = 2 mice were used in each independent experiment, and each independent experiment was repeated at least 3 times. Error bars represent ± SDs, *p < 0.05, **p < 0.01, ***p < 0.001, ****p < 0.0001 by Student’s t test, ^#^p < 0.05 ^##^p < 0.01, ^###^p < 0.001, ^####^p < 0.0001.

The results of the bioinformatics analysis, expression profiling, and functional testing discussed above all suggest that the two populations are epistatic during adipocyte lineage progression. To further test this model, we performed time course studies following induction of adipogenesis. Prior published work established that the first 24–48 h after induction of adipogenesis is a critical interval when progenitor cells commit to progressing down a differentiation pathway.^24^ Consistent with this, we confirmed that the expression levels of *Dpp4*, the previously described marker of interstitial progenitor cells,^14^ decline during this time frame postinduction of adipogenesis (Figure 3A). We then monitored the levels of the markers of the newly identified populations. First, we performed FACS analysis and discovered that stimulation with an adipogenesis differentiation cocktail induces a transition in the cells with the emergence of a significant number of cells in the ICAM1^+^ CD44^low^ population concomitantly with a decline in the number of ICAM1^+^ CD44^high^ cells (Figures 3B and 3C). Next, we monitored these changes in CD44 and the other markers of these populations at the mRNA expression level by performing a time course followed by qRT-PCR. We discovered that the expression markers of the ICAM^+^ CD44^high^ population peak rapidly (within 0.5–6 h) after induction of adipogenesis and then decline to a nadir by 24–48 h (Figure 3D). Reciprocally, expression markers of the ICAM1^+^ CD44^low^ population have a nadir immediately after stimulation and then peak during the 24–48 h time interval (Figure 3E). Together with our prior findings, the results of these experiments indicate that, during differentiation, adipocyte progenitors transition from an ICAM1^+^ CD44^high^ status into an ICAM1^+^ CD44^low^ population. Given the molecular and functional distinctions of these populations, we refer to the ICAM1^+^ CD44^high^ *Noct*-expressing population as Pro-Preadipocytes (pro-PreAs), reserving the designation of PreAs for the ICAM1^+^ CD44^low^ *Txnip*-expressing population that has enhanced activation of PPARγ signaling as well as a notably lower threshold to complete differentiation into adipocytes.

### Noct expression is necessary and sufficient for the pro-PreA expression profile

**Figure 4.**
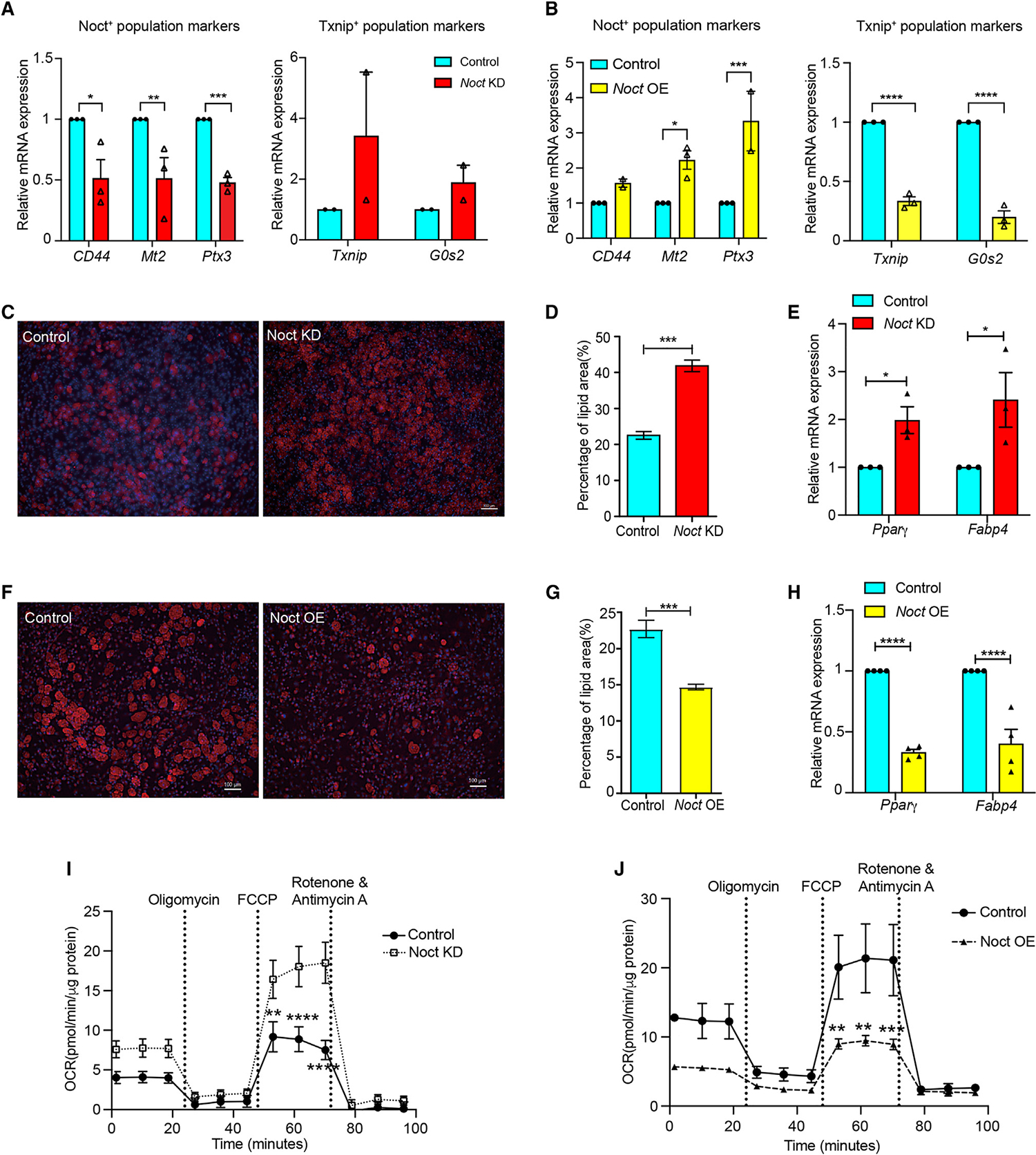
Nocturnin is necessary and sufficient for pro-preadipocyte expression profile (A) qRT-PCR quantifying the expression markers of ICAM^+^ CD44^high^ and ICAM^+^ CD44^low^ populations after knockdown of *Noct*. Significance was determined by Student’s t test. (B) qRT-PCR quantifying the expression markers of ICAM^+^ CD44^high^ and ICAM^+^ CD44^low^ populations after overexpression of *Noct*. Significance was determined by Student’s t test. (C) Knockdown of *Noct* promotes progression through adipogenesis, monitored by BODIPY staining of lipids that mark differentiated adipocytes. (D) Quantification of BODIPY stained lipid area. Quantification was performed using 4 or more images for each condition. Significance was determined by Student’s t test. (E) qRT-PCR quantifying the expression levels of markers of adipogenesis. Significance was determined by Student’s t test. (F) Overexpression of *Noct* inhibits progression through adipogenesis, monitored by BODIPY staining of lipids that mark differentiated adipocytes. (G) Quantification of BODIPY-stained lipid area. Quantification was performed using 4 or more images for each condition. Significance was determined by Student’s t test. (H) qRT-PCR quantifying the expression levels of markers of adipogenesis. Significance was determined by Student’s t test. (I) Seahorse real-time tracing demonstrates that knockdown of *Noct* increases the cellular OCR. Significance was determined by ANOVA with a post hoc Šidák correction. (J) Seahorse real-time tracing demonstrates that overexpression of *Noct* decreases the cellular OCR. Significance was determined by ANOVA with a post hoc Šidák correction. n = 3–6 per condition, Error bars represent ± SDs, *p < 0.05, **p < 0.01, ***p < 0.001, ****p < 0.0001.

Next, we wondered if the expression of any of the population markers we discovered were directly relevant to the functional distinctions we revealed. We were particularly intrigued by the robust differences in *Noct* expression between the pro-PreA and PreA populations (Figure 1J). We further noted that this high expression level significantly dropped in synchrony with the induction of adipogenesis (Figure 3D). In addition, Noct was implicated in adipogenesis and adipose biology in other contexts.^25^ Therefore, we speculated that Noct not only was an expression marker of pro-PreAs but that expression of *Noct* is also integral to the identity of this population. To test this hypothesis, we interrogated the effect of overexpressing and knocking down *Noct* (Figure S3B). Strikingly, when we knock down *Noct*, expression of the other markers of the ICAM1^+^ CD44^high^ pro-PreA population decreased, and expression markers of the ICAM1^+^ CD44^low^ PreAs increased (Figure 4A), indicating that expression of *Noct* is necessary to maintain pro-PreAs. Reciprocally, we found that overexpression of *Noct* is sufficient to induce the distinct expression profile of ICAM1^+^ CD44^high^ pro-PreAs (Figure 4B). Further, overexpression of *Noct* resulted in decreased expression of the markers of the ICAM1^+^ CD44^low^ PreA population (Figure 4B).

We next reasoned that if Noct is necessary and sufficient for the pro-PreA population, then modulating the expression levels of *Noct* would have functional consequences for adipocyte progenitor cells. Indeed, we discovered that knockdown of *Noct* promotes differentiation (Figures 4C–4E), and, reciprocally, overexpression of *Noct* inhibits adipogenesis (Figures 4F–4H) and is analogous to the adipogenicity potential we observed in prospectively isolated pro-PreA and PreA populations (Figures 2F and 2G).

As our above results indicate that NOCT impacts differentiation and *Noct* is a central gene expressed in the pro-PreA population, we hypothesized that *Noct* may be a mechanistic key for the identity or differentiation of this population. *Noct* was previously revealed to regulate cellular metabolism.^26^ In addition, other prior studies indicate that oxygen consumption increases during adipogenesis; furthermore, reducing mitochondrial respiration inhibits differentiation, implicating a functional role for cellular energetics in the process of adipogenesis.^27,28^ Therefore, we tested if manipulating *Noct* expression levels alters the cellular energetics in primary adipose progenitor cells. We used Seahorse technology to quantify the effect of manipulating *Noct* expression levels on the cellular oxygen consumption rate (OCR). These studies revealed that knockdown of *Noct* dramatically increases the maximal respiratory capacity (Figure 4I), and, reciprocally, overexpression of *Noct* results in a lower OCR (Figure 4J). These results support a model where the pro-PreA population maintained by Noct is enforced by regulation of cellular metabolism.

Finally, as we detected evidence that the pro-PreA adipose progenitor population is present in human adipose tissue (Figure 1K), we tested if the effects of manipulating human *NOCT* (*hNOCT*) levels is also conserved. For these studies, we purchased primary human stromal vascular cells, isolated from subcutaneous fat biopsies, and knocked down *hNOCT* expression (Figure S4A). We discovered that decreasing *hNOCT* expression decreases the expression of the pro-PreA population markers *hCD44, hMT2*, and *hPTX3* and, reciprocally, increases the expression of population marker *G0S2* (the change in *hTXNIP* was not significant) (Figure S4A), analogous to what we observed in our mouse studies. At the functional level, as we observed in our studies using mouse adipocyte progenitor cells, we found that decreasing *hNOCT* expression levels in human adipose stromal vascular cells results in an increased OCR (Figure S4B). Together, these results support that at least some of the effects of Noct we discovered in mouse adipose progenitor cells are conserved in human cells.

## DISCUSSION

The above studies elucidate that a population, previously described under the broader category of PreAs, is composed of two populations with closely related but distinguishing expression profiles. Our results support that these populations are epistatic in the adipogenesis pathway and define distinct intermediate cell states in the progression toward the formation of mature adipocytes that are discretely necessary to complete differentiation. These populations can be prospectively isolated from adipose SVF by FACS using the surface markers ICAM and CD44. Expression profiling can also distinguish these populations. It is notable that the surface markers and expression profiling can be monitored during adipogenesis and reveal cells transitioning from one population into the other. We find that distinguishing these populations has functional significance, being necessary for the germane process of adipogenesis, and therefore warrants the introduction of the pro-PreA classifying nomenclature. Our studies also support that Noct might be an identity factor to enforce both the expression profile and the functional properties of pro-PreAs.

### Limitations of the study

Our study focused on the inguinal subcutaneous depot. We elected to focus on this depot because there are robust data from a number of groups detailing a hierarchy of adipocyte precursor cells in this depot that provided the framework and foundation for our current studies. Once analogous underpinnings are established in other adipose depots, it will be feasible, and of interest, to examine those depots for the presence of an equivalent pro-PreA population at both the expression profile and functional levels. For the same reasons, our study focused on a condition that enabled identification of the pro-PreA population. In future studies, it will be of interest to elucidate how different conditions, including aging and altered diets, impact the pro-PreA population. We also note that our studies examined the impact of Noct on adipogenesis. We acknowledge that *Noct* may have additional activities and mechanisms of actions that were not revealed in our studies. Future studies, including the generation of mouse models, where *Noct* expression levels are selectively modulated *in vivo* in adipocyte progenitor cell populations, will require the development of novel genetic tools but hold promise for expanding our understanding of the physiological impact of the role of Noct in pro-PreAs.

## STAR★METHODS

### RESOURCE AVAILABILITY

#### Lead contact

Further information and requests for resources and reagents should be directed to and will be fulfilled by the lead contact, Brian J. Feldman (Brian.Feldman@ucsf.edu)

#### Materials availability

This study did not generate new unique reagents.

#### Data and code availability

- Single-cell RNA-seq data have been deposited in the Gene Expression Omnibus (GEO) under accession number GSE225624. Microscopy data reported in this paper will be shared by the lead contact upon reasonable request.
- This paper does not report original code.
- Any additional information required to reanalyze the data reported in this paper is available from the lead contact upon reasonable request.

### EXPERIMENTAL MODEL AND SUBJECT DETAILS

#### Mice

The C57BL/6J mouse line was obtained from Jackson Laboratories. All animal procedures were performed under the guidance and approval of the University of California (San Francisco) Animal Care and Use Committee. Age and sex are reported in the main text as well as discussed in the limitations of the study section. Mice were maintained in a UCSF vivarium with 12 h light/dark cycles and fed standard chow diets.

Cells: De-identified human subcutaneous preadipocytes were purchased (PT-5020, Lonza). Primary SVF and adipocyte progenitor cells were isolated from male mice as described in STAR Methods below. For adipogenesis assays, cells were cultured in DMEM with 10% FBS in incubators at 37°C with 5% CO_2_.

### METHODS DETAILS

#### FACS to isolate CD31^−^ CD45^−^ cells from SVF

SVF was isolated from the subcutaneous white adipose depots of 8-week old male wild-type C57Bl/6J mice. Briefly, the adipose depots were dissected and then manually minced in DMEM/F12 and digested with collagenase 2 (1.5 units/ml; Roche). They were then incubated at 37°C with agitation for 60 min and vortexing for 10s every 10–15mins. HBSS with 2% FBS was added and the dissociated cells were centrifuged at 350 × g for 5 min. Floating mature lipid-filled adipocytes were aspirated and the cell pellets were resuspended in ACK lysis buffer to remove the red blood cells, followed by the addition of HBSS with 2% FBS. The cells were then filtered through a 40-mM filter and pelleted by centrifugation at 350 × g for 5 min. Cell pellets were resuspended in 200ul FACS staining buffer (5–10 × 10^5^ cells/tube) and incubated with the following antibodies for 30 min at 4°C protected from light. Cells were washed twice with FACS buffer to remove unbound antibodies. The cells were sorted using a BD FACS Aria cell sorter (BD Biosciences) equipped with a 100-μm nozzle and the following lasers and filters: FITC, 488 and 515/20 nm; PE/Cy7, 532 and 780/60 nm. All compensation was performed at the time of acquisition in Diva software by using SVFs for single-color staining, negative staining and fluorescence minus one controls. Gates were set using minus one controls as exemplified in Figure S1D.

#### Immunohistochemistry analysis

Inguinal fat pads were collected and rinsed in cold PBS followed by fixation in 10% neutral buffered formalin for 24 h. The tissues were then dehydrated through a series of graded ethanol treatments and then embedded in paraffin for sectioning. For Nocturnin and ICAM staining, 5μm paraffin sections were deparaffinized and subsequently rehydrated followed by antigen retrieval using citrate buffer. The sections were then incubated with 3% BSA in PBST for 1 h to block nonspecific binding of antibodies. The sections were then incubated with the primary antibodies overnight at 4°C. Sections were then washed and incubated with Alexa Fluor conjugated secondary antibodies (Life Technologies) for 1 h at room temperature followed by mounting medium containing DAPI (Abcam).

#### Single-cell RNA-seq

The CD31^−^ CD45^−^ cells from SVF isolated by FACS (described above) were loaded onto a GemCode instrument (10× Genomics) to generate single-cell barcoded droplets (GEMs) according to the manufacturer’s protocol with the 10× single-cell 3′ v2 chemistry. The libraries were sequenced using an Illumina Novaseq 6000, forward workflow aimed at 250M reads per library sample. Sequence reads were aligned, and gene-level unique molecular identifier (UMI) counts were obtained by using Cell Ranger (Pipeline) in the UCSF Wynton High-Performance Computer Cluster. The Cell Ranger single-cell software suite v.4.0.3 was used to perform sample demultiplexing, alignment, filtering, and UMI counting. The Seurat package (v4.0) on RStudio was used to perforem clustering analysis. Cells were first filtered to have >500 detected genes and less than 5% of total UMIs mapping to the mitochondrial genome. Clusters with very few cells were filtered before downstream analysis. Data were scaled to mitigate the effects of the number of genes detected per cell, the percentage of mitochondrial reads, and the cell cycle phase. Dimensionality reduction and Uniform Manifold Approximation and Projection (UMAP) were performed with Seurat. Seurat was also used for analysis of differential gene expression among the clusters using the FindMarkers function and the Wilcox test. The resolution parameter for the Dimensionality reduction and UMAP was adjusted from 0.1 to 0.35 in this study. Violin plots and individual UMAP plots for the specific genes were performed by Seurat toolkit VlnPlot and FeaturePlot functions, respectively.

#### Adipogenesis assays

SVF and adipocyte progenitor cells were isolated as described above. Cell pellets were resuspended in DMEM with 10% FBS and then filtered through a 40 μm cell strainer. Stroma vascular cells were then used for cell culture. Adipogenesis was induced by culturing 100% confluent cells in DMEM containing 10% FBS (GemCell), 0.5 mM 3-isobutyl-1-methylxanthine (Sigma), 1mM dexamethasone (Sigma) and 5 μg/ml insulin (Sigma) for 2 days and then the cell culture was changed to maintenance media containing 5 μg/ml insulin for 6 days. Minimal differentiation stimulation was with 5 μg/ml of insulin (Sigma).

#### Lipid quantification in adipogenesis assays

Lipid quantification was performed using methods described by Merrick et al.^14^ Briefly, at the end of the adipogenesis assays, cells were stained with BODIPY to label intracellular lipids. Using ImageJ, images were separated into their component channels. Gaussian blur followed by automated thresholding was applied. Then watershed separation of the image was used to quantify the percentage of BODIPY/Lipid stained area.

#### Adipogenic and stemness score calculation

We used Seurat’s AddModuleScore function to calculate module scores for adipogenic and stemness feature expression for the ICAM^+^ CD44^high^ and ICAM^+^ CD44^low^ populations. The approach used, based on,^15,20^ calculates a score reflecting the cell density and expression levels of each category gene set within a population. Adipogenic gene set: Pparγ, Fabp4, Plin2, Lpl, Cebpα, Cd36, Pdgfrβ. Stemness gene set: Ly6a, Pdgfrα, Pi16, Cd34, Itgb1, Cd55, Wnt2, Anxa3, Cebpβ, Ebf2, Fn1.

#### Pseudotime analysis

Pseudotime analysis was performed using the R package Monocle2. Cell types were annotated using Seurat, imported into Monocle2 and a single-cell trajectory was constructed using the default settings. Cell’s local density (rho) and nearest distance (delta) were used to determine the number of clusters. The top 1000 differentially expressed genes were used as the ordering genes, implemented DDRTree for dimension reduction and performed the trajectory analysis.

#### Isolation of RNA and RT-qPCR

Total cellular RNAs were purified using RNeasy Lipid Tissue Mini kit or RNeasy Plus Mini Kit (Qiagen) according to manufacturer’s instructions. DPP4, ICAM1+ CD44^high^ and ICAM1+ CD44^low^ cells were isolated by FACS directly into RLT buffer and total RNA was isolated using RNeasy Plus Micro Kit (Qiagen) according to the manufacturer’s protocol. RNA concentration and quality were measured using Nano-drop 2000 (Thermo Fisher Scientific) and a Bioanalyzer 2100 Eukaryote Total RNA Nano kit (Agilent; Santa Clara, CA). At least four independent samples were prepared for each experiment and all samples had an RNA integrity number (RIN) greater than 7. Equal amounts of RNA were reverse transcribed using iScript cDNA Synthesis kit (BioRad) following the manufactures protocol. RT–qPCR was performed using a Biorad CFX96 Real-Time PCR System. All real-time qPCR reactions were carried out with technical triplicates. A list of the primers used is in Table S1.

#### Human subcutaneous preadipocytes

Human subcutaneous preadipocytes were purchased (PT-5020, Lonza) and differentiated according to the manufacturer’s protocol. Briefly, confluent preadipocyte were treated with Preadipocyte Growth Medium-2 (PGM-2TM) (PT-8202, Lonza) containing 10% FBS, 2 mM glutamine, IBMX (1:1000, PT-9502, Lonza), indomethacin (1:500, PT-9502, Lonza), dexamethasone (1:1000, PT-9502 Lonza) and insulin (1:100, PT-9502, Lonza). Confluent cells in 48-well plate were transfected with 7.5 pM sihNOCT (Thermofisher, 1299001) or siContrl (Santa Cruz, sc-37007) for 24 h and then differentiated for 2 days before being used in Seahorse assays or lysed for RNA extraction and RT-qPCR analysis. A list of the primers used is in Table S1. For human cell Seahorse XF Cell mito stress test, cells were directly seeded into XFe24 cell culture microplates (V7).

#### Seahorse XF cell mito stress test

Subcutaneous SVFs were isolated from 8 week wild-type mice and plated into 24-well plates. After 24hrs, cells were transfected with Nocturnin plasmid pCMV-mNoc-3X FLAG (Addgene plasmid 90465) with Lipofectamine 2000 (Invitrogen) or siNOCT(ThermoFisher, catalog 4390071) with Lipofectamine RNAiMAX (ThermoFisher) and cultured for another 24 h. Cells were transferred into XFe24 cell culture microplate (Agilent) for 24hrs. Prior to placing the plates into the Seahorse, the cells were rinsed with Seahorse XF DMEM Medium (Agilent) containing 1mM Sodium Pyruvate, 2mM Glutamax, 7mM Glucose and pre-incubated in a non-CO_2_ incubator for 1h at 37°C. The oxygen consumption rate (OCR) in the cultured cells was measured using the Seahorse XFe Extracellular Flux Analyzer (Agilent). Cells were subjected to a mitochondrial stress test by adding oligomycin at a final concentration of 1.0 μM, carbonyl cyanide 4-(trifluoromethoxy)phenylhydrazone (FCCP) of 4 μM and rotenone/actinomycin A of 1 μM. The OCR measurements were conducted with 3 assay cycles: mix 3 min, wait 2 min and measure 2 min for each compound. OCR was normalized to the amount of protein in each well. Data were analyzed using Agilent Seahorse Analytics.

### QUANTIFICATION AND STATISTICAL METHODS

Statistical methods were not used to predetermine sample size. The experiments were not randomized, and investigators did not perform the experiments blinded. For comparisons between two independent groups, a Student’s t test was used and p < 0.05 was considered statistically significant. For multiple comparisons ANOVA was performed with post hoc correction. All statistical analyses were performed with Prism Graphpad software.

## Supplementary Material

1

## Figures and Tables

**KEY RESOURCES TABLE:** 

REAGENT or RESOURCE	SOURCE	IDENTIFIER

Antibodies		

PE/Cyanine7 anti-mouse/human CD44 Antibody	Biolegend	Cat#103030; RRID:AB_830787
FITC anti-mouse CD26 (DPP-4) Antibody	Biolegend	Cat#137806; RRID:AB_10663402
PE/Cyanine7 anti-mouse CD54(Icam1)Antibody	Biolegend	Cat#116122; RRID:AB_2715950
APC anti-mouse CD31 Antibody	Biolegend	Cat#102410; RRID:AB_312905
APC anti-mouse CD45 Antibody	Biolegend	Cat#103112; RRID:AB_312977
Anti-Mouse CD31 (PECAM-1) FITC	eBioscience/Thermo-Fisher	Cat#11–0311-81; RRID:AB_465011
7-AAD	BD Bioscience	Cat#559925: RRID:AB_2869266
ICAM-1/CD54 Antibody (009) for IHC	Novus Biologicals	Cat#NBP2–90585
Nocturnin Antibody (F-4) for IHC	Santa Cruz Biotechnology	Cat#376584; RRID:AB_11149724
Donkey anti-Rabbit IgG (H + L) Highly Cross-Adsorbed Secondary Antibody, Alexa Fluor^TM^ 488	Thermo-Fisher	Cat#A-21206; RRID:AB_2535792
Goat anti-Mouse IgG (H + L) Highly Cross- Adsorbed Secondary Antibody, Alexa Fluor^TM^ 594	Thermo-Fisher	Cat#A-11032; RRID:AB_2534091

Biological samples		

Human Subcutaneous Preadipocyte Cells	Lonza	Cat#PT-5001

Chemicals, peptides, and recombinant proteins		

Lipofectamine RNAiMAX Transfection Reagent	Thermo-Fisher	Cat#13778100
BODIPY 558/568 C12	Thermo-Fisher	Cat#D3835
TaqMan Gene Expression Master Mix	Thermo-Fisher	Cat#4369016
RNeasy Mini Kit	Qiagen	Cat#74106
iScript Reverse Transcription Supermix	Bio-Rad	Cat#1708841
Seahorse XF Cell Mito Stress Test Kit	Agilent	Cat#103015–100
Seahorse XF DMEM Medium, pH 7.4	Agilent	Cat#103575–100

Deposited data		

scRNA-Seq Data Files	Gene Expression Omnibus (GEO)	GSE225624

Experimental models: Organisms/strains		

C57Bl/6 mice	Jackson Laboratories	Cat#000664

Oligonucleotides		

TaqMan Gene Expression Assays	See Table S1 for list of TaqMan primers	

Recombinant DNA		

Nocturnin siRNA ID s63547	Thermo-Fisher	Cat#4390771
Nocturnin siRNA (mouse)	Santa Cruz Biotech	Cat#62698
Txnip siRNA (siRNA IDMSS226056)	Thermo-Fisher	Cat#1320001
Control siRNA-A	Santa Cruz Biotech	Cat#37007

Software and algorithms		

R and R studio	CRAN	R-4.2.3 https://cran.r-project.org/bin/windows/base/
Prism	Graphpad	Prism 8 https://www.graphpad.com/features
Flowjo	BD Biosciences	Flowjo_V10 https://www.bdbiosciences.com/en-us/products/software/flowjo-v10-software
Illustrator	Adobe	
FIJI_ImageJ	FIJI	https://fiji.sc/
Seurat	Satijalab	https://satijalab.org/seurat/
Monocle	Trapnell-lab	http://cole-trapneN-lab.github.io/monocle-release/

## Highlights

- Identification of a pro-preadipocyte population in mouse and human white adipose tissue
- Pro-preadipocytes are ICAM1^+^ CD44^high^ *Noct*-expressing cells
- *Noct* modulates adipogenesis and enforces a pro-preadipocyte expression profile
