# Supplementary material for: Identification of an adipose tissue-resident pro-preadipocyte population
Source: Cell Rep. Author manuscript; Available in PMC 2023 Jul 26. (PMC10370484; doi:10.1016/j.celrep.2023.112440)
Supplement: 1 [file NIHMS1905360-supplement-1.pdf]

Cell Reports, Volume *42*

Supplemental information

Identification of an adipose tissue-resident pro-preadipocyte population

Min Chen, Soochi Kim, Liang Li, Sourav Chattopadhyay, Thomas A. Rando, and Brian J. Feldman

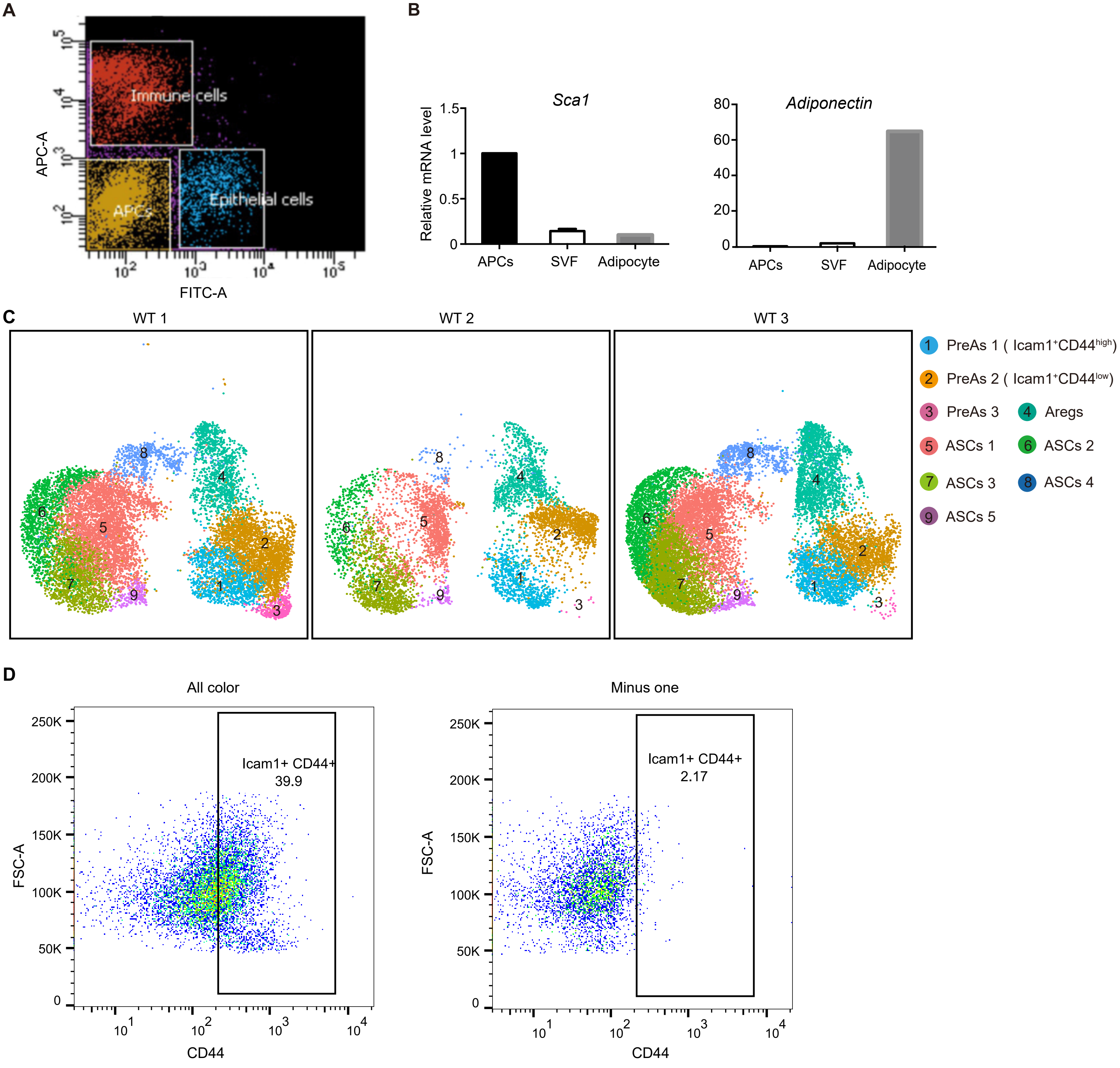

Figure S1: Isolation and analysis of CD31- CD45- cells from the SVF. Related to Figure 1.

(A) Representative FACS plot with gates used to isolate CD31$^-$ CD45$^-$ (APC) cells.

(B) RT-qPCR measuring Sca1 and adiponectin levels in CD31$^-$ CD45$^-$ (APCs) compared to SVF and mature adipocytes confirming that the FACS isolated CD31$^-$CD45$^-$cells selectively and specifically express high level of *Sca1* and undetectable levels of Adiponectin.

(C) UMAP analysis of the CD31$^-$ CD45$^-$ cells isolated from independent mice demonstrating reproducible results across the biological replicates.

(D) Representative cytometry plots from objective gate setting. By comparing all color staining with minus one controls, gates were established for cytometry and FACS.

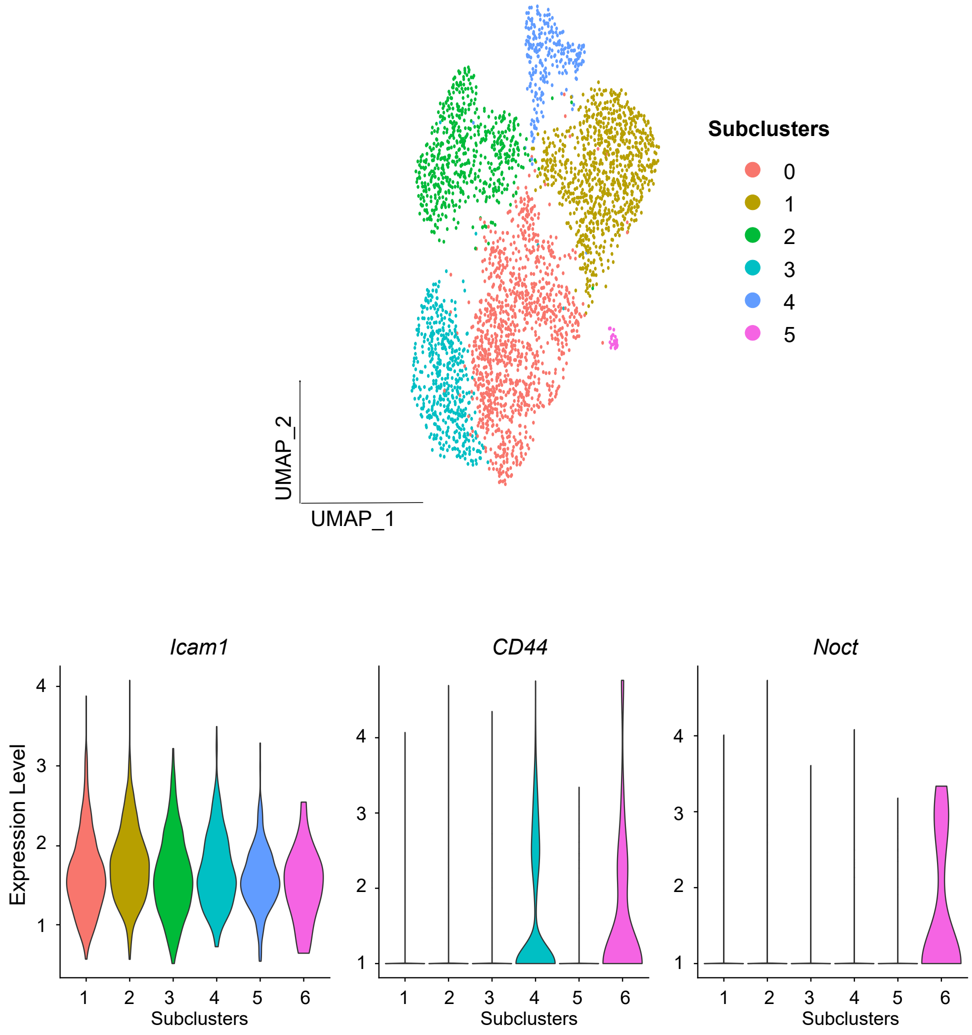

Figure S2: Icam^{+} CD44^{high} *Noct* expressing population of cells is present in adipose tissue publicly available datasets. Related to Figures 1 and 2.

(Upper) UMAP of Icam1+ cells from SCP1376 mouse adipose stem and progenitor cell subcluster data (Emont et al., 2022), with 6 distinct subclusters. (Lower) Violin plots highlighting subcluster 5 contains a Icam^{+} CD44^{high} *Noct* expressing population.

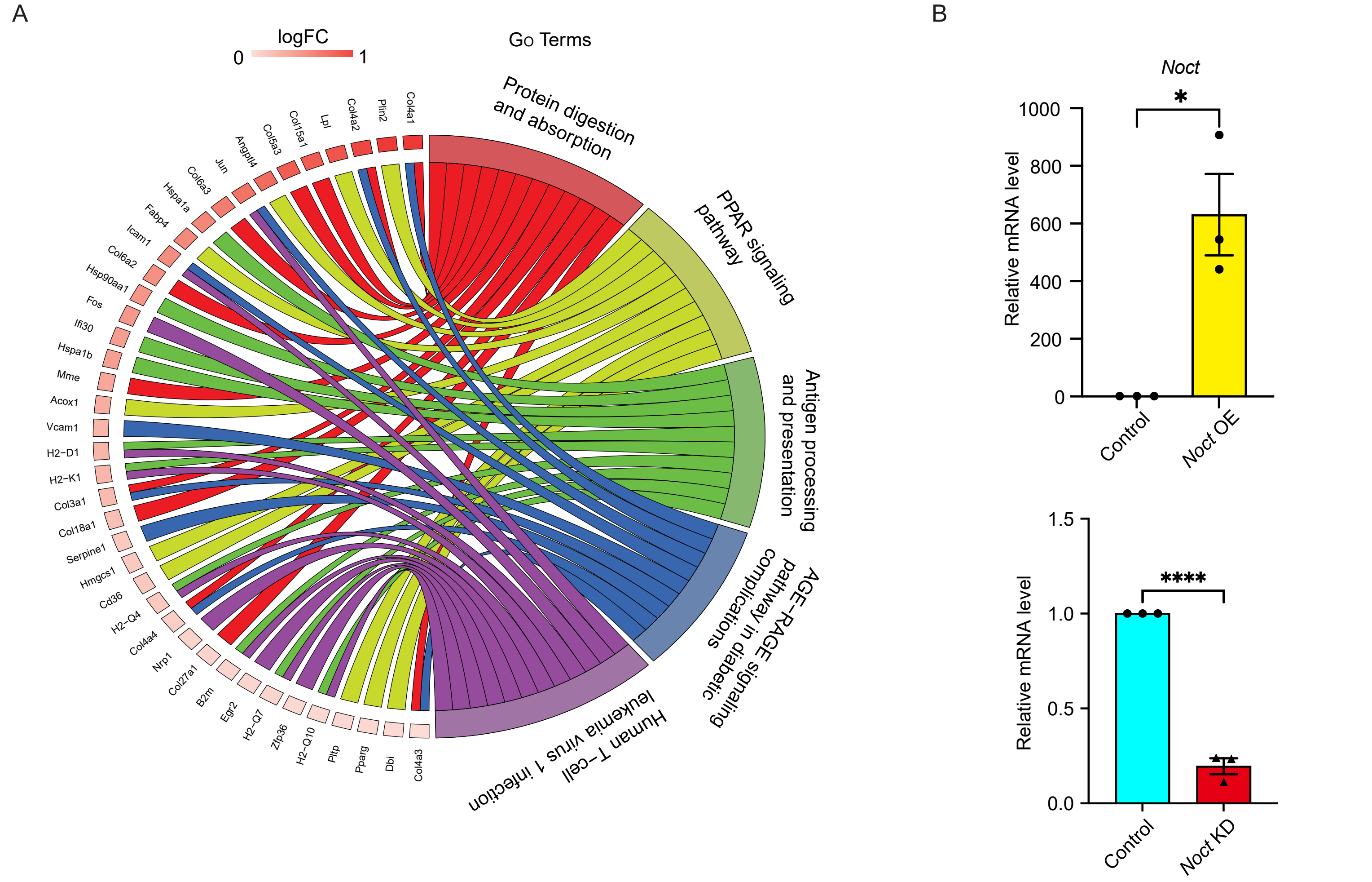

Figure S3: PPAR signaling distinguishes pro-preadipocytes and preadipocytes and expression of Nocturnin is necessary and sufficient for the pro-preadipocyte expression profile. Related to Figure 2.

(A) Gene ontology (GO) analysis of the ICAM1^{+} CD44^{high} Txnip^{+} population reveals distinct enrichments, including enhanced Pparg activity.

(B) RT-qPCR demonstrating efficient knockdown and overexpression of Noct in adipose progenitor cells.

Significance as determined by Student’s t-test. Error bars represent ± SDs, *p<0.05, ****p<0.0001

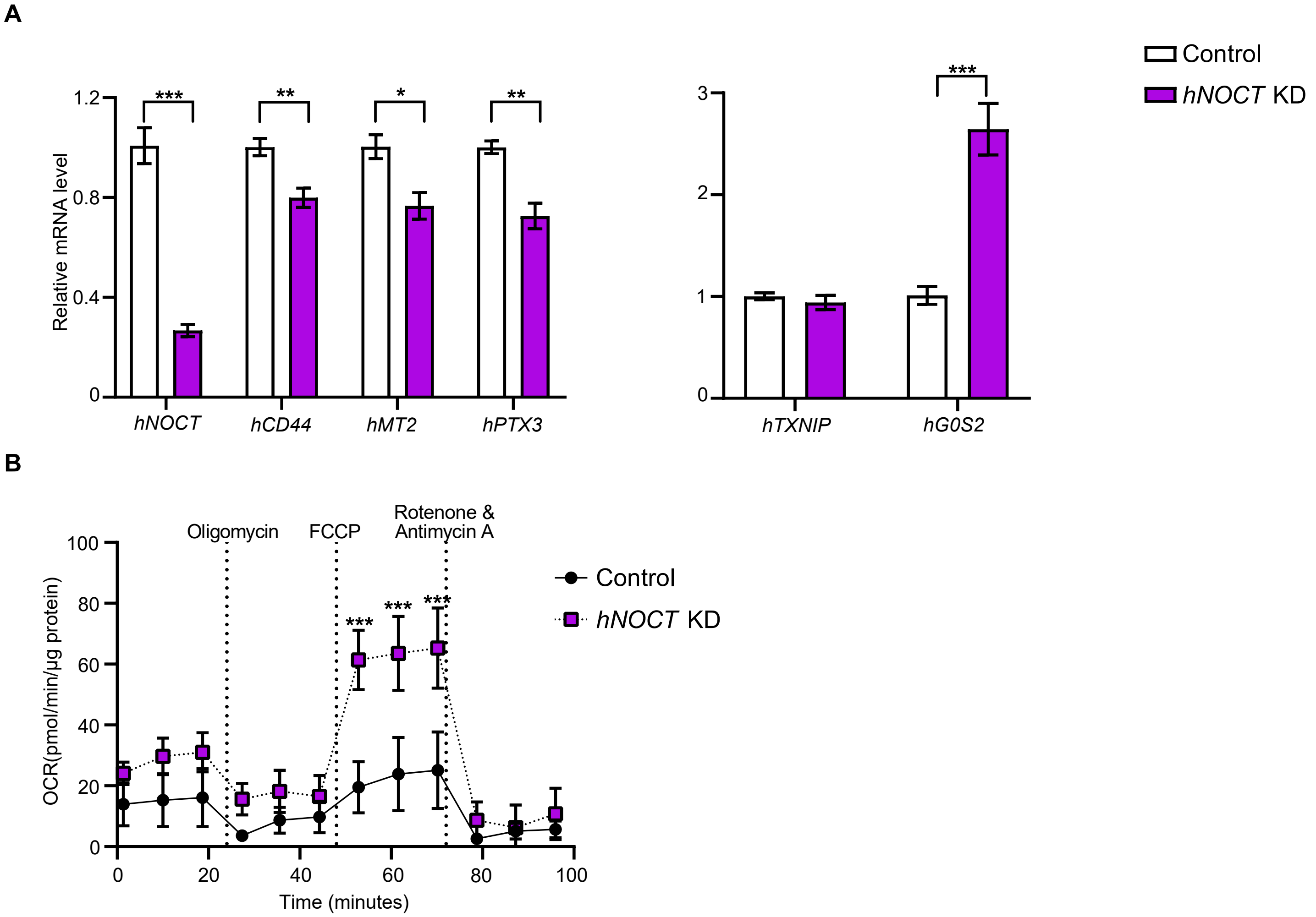

Figure S4: Effects of *hNOCT* are conserved in human stromal vascular cells obtained from subcutaneous fat pad biopsies. Related to Figure 4.

(A) RT-qPCR quantifying the expression levels of human homologues of the mouse markers of pro-preadipocytes and preadipocytes after knockdown of *hNOCT* expression. Significance was determined by Student's t-test.

(B) Seahorse real-time tracing demonstrates that knockdown of *hNOCT* increases the cellular OCR. Significance was determined by ANOVA with a a post hoc Šidák correction.

Error bars represent ± SDs N=3-4 per condition, Error bars represent ± SDs, *p<0.05, **p<0.01, ***p<0.001

Table S1: List of primers. Related to STAR Methods

Mouse Primer Name	Source
Txnip (Mm01265659_g1)	Thermo-Fisher
Noct (Mm00802276_m1)	Thermo-Fisher
Ptx3 Mm00477268_m1)	Thermo-Fisher
Mt2 (Mm00809556_s1)	Thermo-Fisher
Cd44 (Mm01277163_m1)	Thermo-Fisher
TBP (Mm00446971_m1)	Thermo-Fisher
GOs2 (Mm00484537_g1)	Thermo-Fisher
C/ebpa (Mm00514283_s1)	Thermo-Fisher
Fabp4 (Mm00445878_m1)	Thermo-Fisher
Pparg (Mm00440940_m1)	Thermo-Fisher
**Human Primer Name**	
FABP4_Human (Hs01086177_m1)	Thermo-Fisher
PPARG_Human (Hs01115513_m1)	Thermo-Fisher
TBP_Human (Hs00427620_m1)	Thermo-Fisher
Nocturnin_Human (Hs00232597_m1)	Thermo-Fisher
Mt2_Human (Hs01591333_g1)	Thermo-Fisher
CD44_Human (Hs01075864_m1)	Thermo-Fisher
Ptx3_Human (Hs00173615_m1)	Thermo-Fisher
Txnip_Human (Hs01006897_g1)	Thermo-Fisher
G0s2_Human (Hs00377852_g1)	Thermo-Fisher
